# Dye Displacement Assay for Saccharides using Benzoxaborole Hydrogels

**DOI:** 10.1002/open.201700193

**Published:** 2018-01-30

**Authors:** Emma V. Lampard, Adam C. Sedgwick, Thitima Sombuttan, George T. Williams, Boontana Wannalerse, A. Toby A. Jenkins, Steven D. Bull, Tony D. James

**Affiliations:** ^1^ Department of Chemistry University of Bath BA2 7AY Bath UK; ^2^ The Department of Chemistry, Faculty of Science Kasetsart University 50 Ngam Wong Wan Road, Lat Yao, Chatuchak Bangkok 10900 Thailand; ^3^ The Center of Excellence for Innovation in Chemistry, Faculty of Science Kasetsart University 50 Ngam Wong Wan Road, Lat Yao, Chatuchak Bangkok 10900 Thailand

**Keywords:** boronic acids, colorimetric detection, dye displacement assays, glucose, saccharides

## Abstract

Dye displacement assays are a simple but effective method to determine the concentration of target analytes. Previously, we have shown that phenylboronic acid pinacol ester hydrogels (borogels) can be used to develop a boronic acid–Alizarin red S dye displacement assay for the determination of fructose (orange to red). In this work, benzoxaborole hydrogels (BOBgels) were developed, and these BOBgels demonstrated an enhanced apparent binding affinity towards monosaccharides, in particular towards glucose.

In dye displacement assays, the dye is reversibly bound to a specific receptor. The addition of a competitive analyte results in the displacement of the dye from the host, eliciting a response as an optical signal. These systems have several advantages over traditional sensing assays, which include a non‐covalently bound dye, enabling the use of different dyes on the same receptor and the system works well in both aqueous and organic solvents. Owing to this, these systems have been elegantly employed by many research groups.^1^, ^2^, ^3^, ^4^, ^5^, ^6^, ^7^

Boronic acids have a well‐known affinity to bind to 1,2‐ and 1,3‐diols.^8^, ^9^ Therefore, boronic acids in combination with the (1,2‐diol containing) dye Alizarin red S (ARS) have been developed into dye displacement assays for the detection of various analytes.^10^, ^11^, ^12^ Previously, we developed an ARS–boronate hydrogel (borogel) displacement assay by utilizing the strong affinity of boronic acids towards saccharides (Scheme 1).^13^ Prior to treatment with ARS, the borogel appeared colorless (including blank); however, the addition of ARS resulted in red (blank) and orange borogels. The visual color change of red to orange for ARS is indicative of its binding to boronic acids. It was then shown that the addition of fructose resulted in the displacement of the ARS dye, providing a measurable increase in absorbance at 513 nm in solution. Thereby resulting in a method for the determination of the concentration of saccharides in solution.

**Scheme 1:**
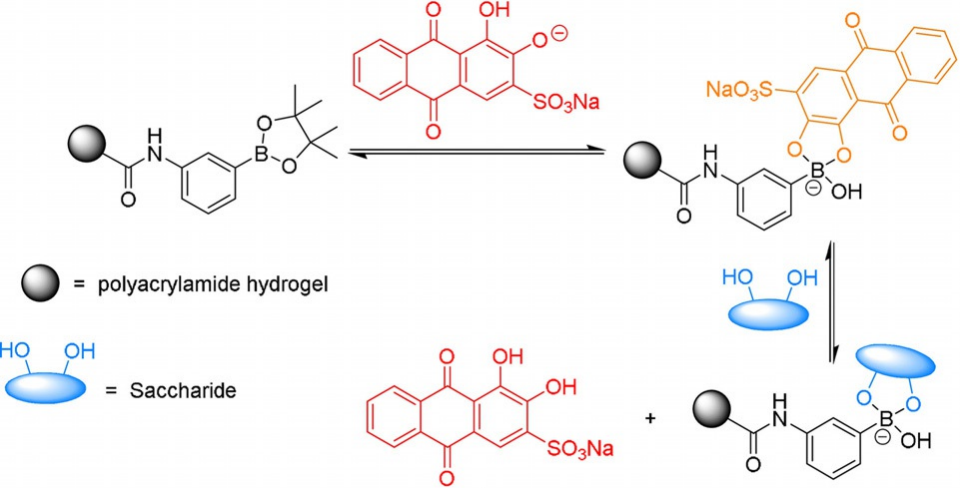
Previously reported dye displacement assay utilizing an ARS‐bound borogel for the determination of fructose concentration.

Despite boronic acids being regarded as one of the best receptors for the recognition of carbohydrates in water,^9^ they have a number of limitations. These include the inability to bind non‐reducing sugars and glycosides, which account for a large proportion of biologically important oligosaccharides. However, Dowlut and Hall reported that *ortho*‐hydroxyalkyl arylboronic acids (benzoxaboroles) were capable of binding to glycosides.^14^ Benzoxaboroles were also shown to bind to monosaccharides such as fructose and glucose with higher affinity compared to other boronic acids in neutral water, and also displayed an enhanced solubility profile.^14^

Therefore, in this research, we turned our attention to the development of borogels containing benzoxaboroles. To afford the benzoxaborole monomer (BOB), 2‐formylphenylboronic acid was treated with NaBH_4_, reducing the aldehyde functionality. The generated alcohol immediately underwent cyclization to form the oxaborole ring with the loss of a molecule of water. The BOB intermediate was subsequently treated with fuming nitric acid to afford 6‐nitrobenzoxaborole in good yield (66 %). 6‐Nitrobenzoxaborole was then reduced by using hydrogenation conditions (Pd/C), giving 6‐aminobenzoxaborole in good yield (78 %). The methylacrylamide BOB monomer was prepared by the addition of methylacryloyl chloride to 6‐aminobenzoxaborole in excellent yield (82 %).

With 6‐methacryloylamino BOB monomer in hand, a series of polyacrylamide hydrogels were synthesized, consisting of water (60 % w/w), acrylamide (38 % w/w), methylene bisacrylamide (1 % w/w), and either 6‐methacryloylamino BOB monomer (1 % w/w) or 3‐methacyryloylamino phenylboronic acid pinacol ester monomer (PBA; 1 % w/w). Blank gels were also prepared containing additional acrylamide in the place of the boronate compound (1 % w/w) (Scheme 2).

**Scheme 2:**
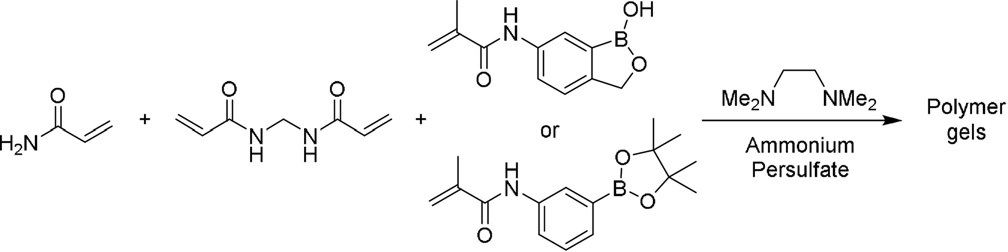
Preparation of boronate ester hydrogels.

For qualitative purposes, treatment of the blank gel, borogel, and BOBgel with ARS solution (pH 7.3) resulted in red (blank) and orange (borogel and BOBgel) slabs. Gel slabs were then placed in PBS to wash out any non‐specifically bound dye. The orange color of the borogels and BOBgels persisted after washing, whereas the blank gel was pale pink, owing to a small amount of non‐specifically bound dye remaining (see the Supporting Information).

To obtain quantitative data, hydrogels were prepared in plastic disposable syringes, which provided a method that consistently produces an identically sized hydrogel. Further to this, the size of the polymer gels can easily be modified by increasing or decreasing the amount of polymer solution taken up into the syringe.

The hydrogel cylinders (0.1 g) were immersed in 2.0×10^−4^ m ARS solution [in phosphate buffer solution (PBS)]. As illustrated in Figure 1, the decrease in absorbance at 513 nm corresponds to the amount of dye uptake into the gel. After 5 h, both borogel and BOBgel were completely dye saturated with no further decrease in absorption at 513 nm. Similar to the gel slabs, both borogels and BOBgels appeared orange in color, whereas the blank gels were red.

**Figure 1:**
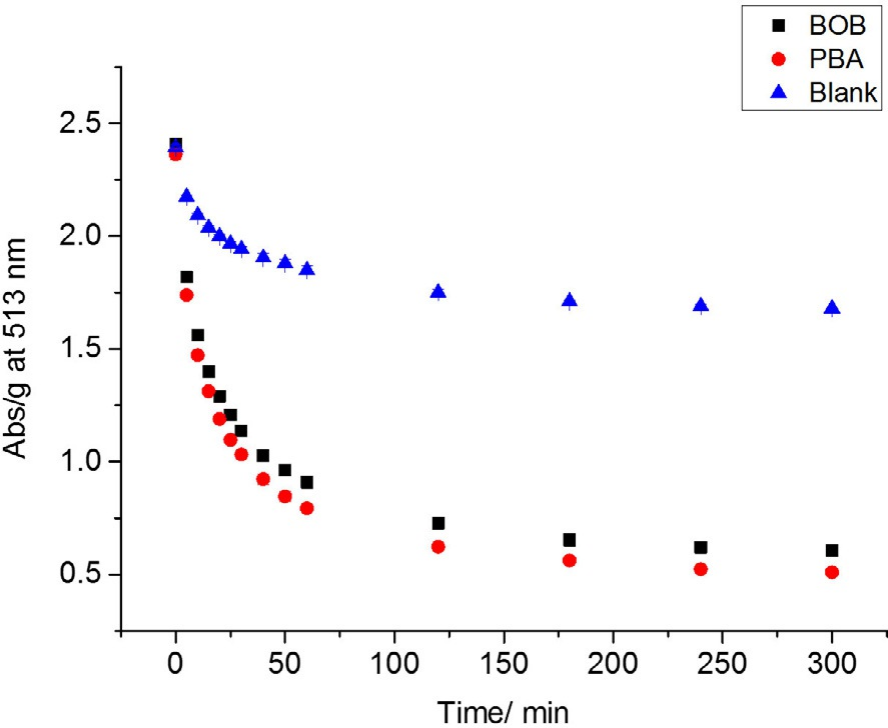
UV absorption (at 513 nm) measurements for dye (ARS) uptake per unit mass of gels versus time for smaller hydrogel cylinders in PBS solution (pH 7.3).

Each gel was then placed in PBS to wash out any non‐boron‐bound dye. As previously reported, there was no further increase in dye concentration in solution after 2 h (see the Supporting Information).

The PBS‐washed hydrogels were then exposed to an increasing concentration of glucose (0–1 m). As shown in Figure 2, an increase in the addition of glucose led to the displacement of ARS from the borogel, leading to an increase in absorption at 513 nm. The largest amount of dye release was seen for the BOBgels, indicating a greater affinity for saccharides over the simple boronate receptor—borogel. Scheme 3 illustrates the dye displacement assay for the binding of a saccharide to BOB, displacing the ARS dye.

**Figure 2:**
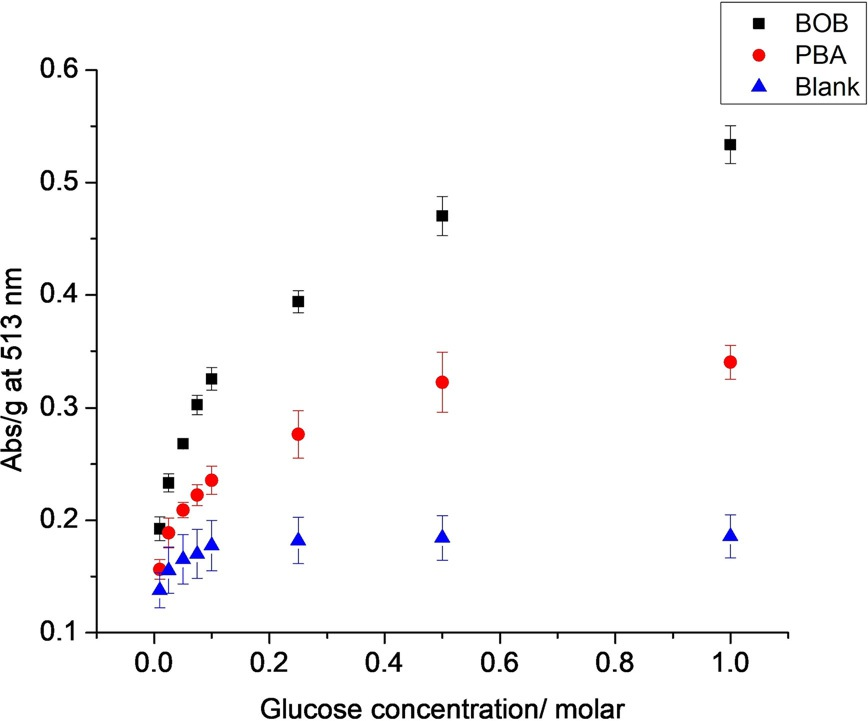
UV absorption (at 513 nm) measurements for glucose addition to ARS dye displacement assay for smaller hydrogel cylinders in PBS solution (pH 7.3).

**Scheme 3:**
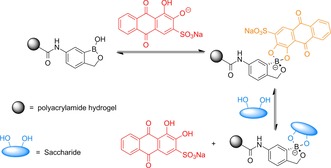
An improved dye displacement assay utilizing an ARS‐bound BOBgel for the determination of fructose concentration.

We then turned our attention to the addition of various saccharides to the borogels and BOBgels (Table 1). In comparison to glucose, a similar response was observed for galactose and mannose; however, both the borogels and BOBgels released considerably more dye when exposed to fructose. This is consistent with the saccharide binding stability constants reported for phenylboronic acid by Lorand and Edwards (fructose > other saccharides).^15^ Unfortunately, the addition of methyl α‐d‐glucopyranoside to both borogel and BOBgel resulted in no change in absorbance.

**Table 1:** Amount of dye released [abs g^−1^ at 513 nm] upon the addition of various saccharides (1 m).

Gel	Fructose	Galactose	Mannose	Glucose
BOB	0.94	0.53	0.59	0.53
PBA	0.82	0.40	0.41	0.34
blank	0.23	0.21	0.22	0.19

In summary, borogels and BOBgels have been prepared. Interestingly, BOBgels demonstrated an enhanced apparent binding affinity for all the monosaccharide sugars evaluated. With the greatest enhancement in binding being observed with d‐glucose and the BOBgels, relative to borogels. We are currently exploring the utility of borogels and BOBgels for the detection of biologically important saccharides under a variety of conditions.

## Experimental Section

### General Methods

All starting materials and reagents were purchased from Sigma Aldrich, Alfa Aesar, Fluorochem, Acros Organics, or Apollo Scientific and used as received without any further purification. Unless otherwise stated, all solvents used were reagent grade and were used without distillation. Dry solvents were obtained from an Innovative Technology Inc. PS‐400‐7 solvent purification system. All water was distilled. PBS buffer solution (52.1 wt % MeOH) was prepared according to the literature.^16^ Thin‐layer chromatography was performed by using commercially available Macherey–Nagel aluminum‐backed plates coated with a 0.20 mm layer of silica gel (60 Å) with fluorescent indicator UV254. These plates were visualized by using ultraviolet light with a wavelength of either 254 or 365 nm, or by staining the plates with vanillin or ninhydrin solution. Silica gel column chromatography was carried out by using Fisher or Sigma Aldrich 60 Å silica gel (35–70 μm).

Unless otherwise stated, all NMR spectra were obtained by using a Bruker Advance 300 with all spectra recorded in chloroform‐*d* or [*D*_6_]DMSO. ^1^H NMR spectra were recorded at an operating frequency of 300 MHz, ^11^B NMR spectra were recorded at an operating frequency of 96 MHz, and ^13^C NMR spectra were recorded at an operating frequency of 75 MHz, with proton decoupling for all ^13^C NMR spectra. High‐resolution mass spectrometry (HRMS) results were typically acquired on an externally calibrated Bruker Daltonics micrOTOF time‐of‐flight mass spectrometer coupled to an electrospray source (ESI‐TOF). All solvents used in fluorescence measurements were HPLC or fluorescence grade and the water was deionized. Further reprocessing of the data was carried in OriginPro 8.0 software. All pH measurements taken during fluorescence/absorption experiments were recorded on a Hanna Instruments HI 9321 microprocessor pH meter, which was routinely calibrated by using Fisher Chemicals standard buffer solutions (pH 4.0: phthalate; 7.0: phosphate; 10.0: borate). UV/Vis measurements were performed on a PerkinElmer Lambda 20 Spectrophotometer, utilizing a Starna silica (quartz) cuvette with a 10 mm path length (two faces polished). Further reprocessing of the data was carried out in OriginPro 8.0 software.

### Synthesis of Hydrogel Monomers

Previously reported methacyryloylamino PBA was synthesized according to the literature reported procedure.^13^

See the Supporting Information for a full synthetic procedure of BOB monomer.

## Conflict of interest

*The authors declare no conflict of interest*.

## Supporting information

As a service to our authors and readers, this journal provides supporting information supplied by the authors. Such materials are peer reviewed and may be re‐organized for online delivery, but are not copy‐edited or typeset. Technical support issues arising from supporting information (other than missing files) should be addressed to the authors.

Supplementary
