# Supplementary material for: Dye Displacement Assay for Saccharides using Benzoxaborole Hydrogels
Source: ChemistryOpen. 2018 Jan 30;7(3):266–8. doi: 10.1002/open.201700193 (PMC5838390; doi:10.1002/open.201700193)

ChemistryOPEN

Supporting Information

© 2018 The Authors. Published by Wiley-VCH Verlag GmbH & Co. KGaA, Weinheim

Dye Displacement Assay for Saccharides using Benzoxaborole Hydrogels

Emma V. Lampard,[a] Adam C. Sedgwick,*[a] Thitima Sombuttan,[b] George T. Williams,[a] Boontana Wannalerse,*[b, c] A. Toby A. Jenkins,[a] Steven D. Bull,[a] and Tony D. James*[a]

open_201700193_sm_miscellaneous_information.pdf

Supporting Information

Content

1. **Qualitative data**
2. **Gel swelling studies**
3. **Quantitative data**
4. **Limit of Detection**
5. **Experimental**
6. **NMR**

1. Qualitative data

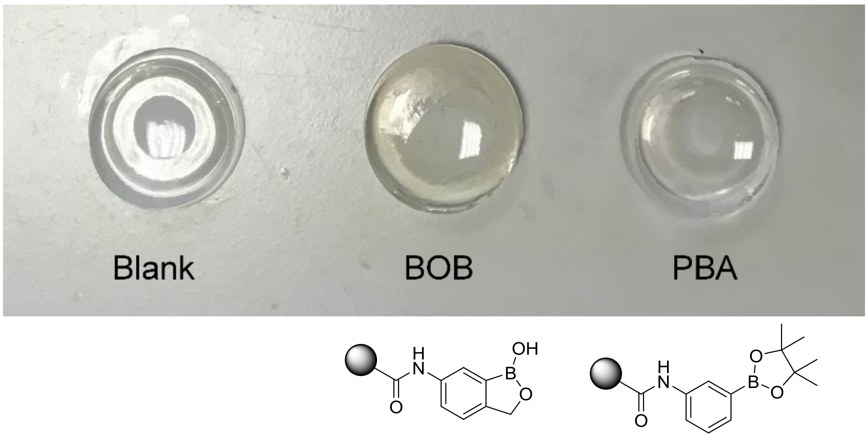

Figure S1 – Photograph of gel slabs, which includes blank hydrogel, BOBgel and borogel

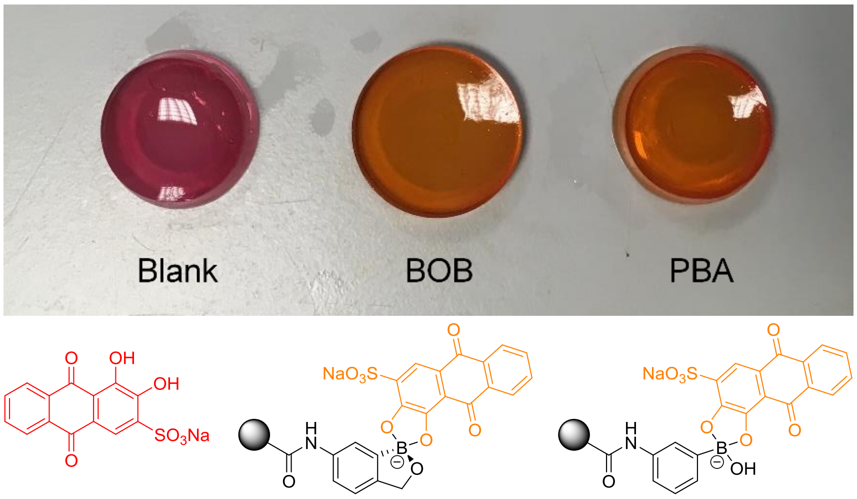

Figure S2 – Photograph of gel slabs after treatment with alizarin red S, which includes blank hydrogel (Red), BOBgel (Orange) and borogel (Orange)

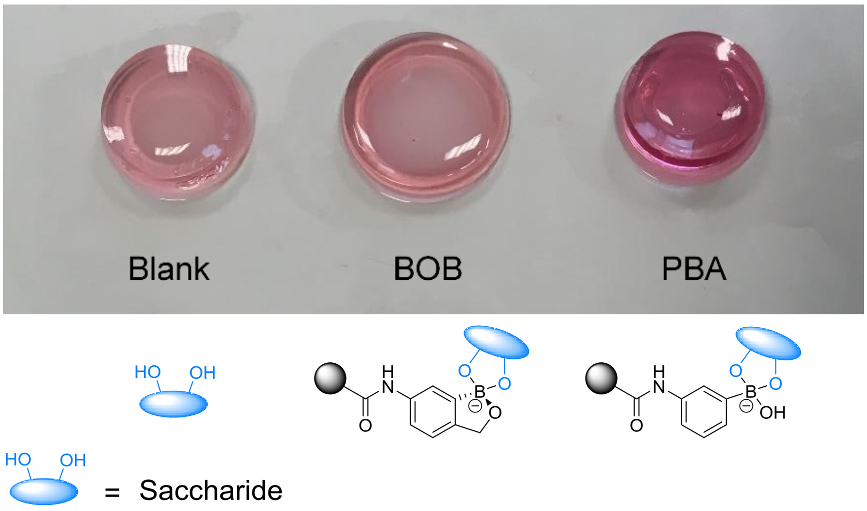

Figure S3 – Photograph of alizarin red S bound gel slabs after treatment with fructose, which includes blank hydrogel (Red), BOBgel (Red) and borogel (Red)

2. Gel swelling studies

In order to investigate factors that could affect the volume of the hydrogels, gel swelling studies were carried out. Blank, benzoxaborole-functionalised and phenylboronic acid pinacol ester-functionalised hydrogels were immersed overnight in pure water, pH 7.3 phosphate buffer, pH 8.5 phosphate buffer, pH 10 phosphate buffer, pH 8.21 phosphate buffer (containing 52.1 wt % methanol) and pH 10 phosphate buffer (containing 52.1 wt % methanol). Equilibrium swelling studies were carried out at room temperature. The hydrogels were weighed before and after standing in various buffers.

Table S1. Percentage mass changes for blank hydrogels after standing in various buffers.

**Buffer**	**Repeat 1 % change**	**Repeat 2 % change**	**Repeat 3 % change**	**Average % change**
Pure water	+36.04	+38.00	+35.91	+36.65
pH 7.3 phosphate	+38.63	+41.51	+38.18	+39.44
pH 8.5 phosphate	+38.17	+42.09	+38.57	+39.61
pH 10 phosphate	+38.68	+41.26	+38.53	+39.49
pH 8.21 phosphate (with MeOH)	-37.34	-37.62	-36.56	-37.17
pH 10 phosphate (with MeOH)	-37.51	-36.48	-36.54	-36.85

Table S2. Percentage mass changes for BOBgels after standing in various buffers.

**Buffer**	**Repeat 1 % change**	**Repeat 2 % change**	**Repeat 3 % change**	**Average % change**
Pure water	+46.57	+45.50	+45.03	+45.70
pH 7.3 phosphate	+43.91	+46.95	+44.46	+45.11
pH 8.5 phosphate	+43.43	+44.82	+43.66	+43.97
pH 10 phosphate	+44.08	+45.81	+45.96	+45.28
pH 8.21 phosphate (with MeOH)	-37.65	-40.84	-38.35	-38.95
pH 10 phosphate (with MeOH)	-37.10	-39.93	-38.50	-38.51

Table S3. Percentage mass changes for phenylboronic acid pinacol ester-functionalised hydrogels (borogels) after standing in various buffers.

**Buffer**	**Repeat 1 % change**	**Repeat 2 % change**	**Repeat 3 % change**	**Average % change**
Pure water	+41.86	+42.55	+41.45	+41.95
pH 7.3 phosphate	+37.46	+40.43	+39.76	+39.22
pH 8.5 phosphate	+39.11	+42.49	+39.16	+40.25
pH 10 phosphate	+39.30	+42.06	+38.69	+40.02
pH 8.21 phosphate (with MeOH)	-35.67	-37.65	-36.96	-36.76
pH 10 phosphate (with MeOH)	-36.08	-35.55	-36.82	-36.15

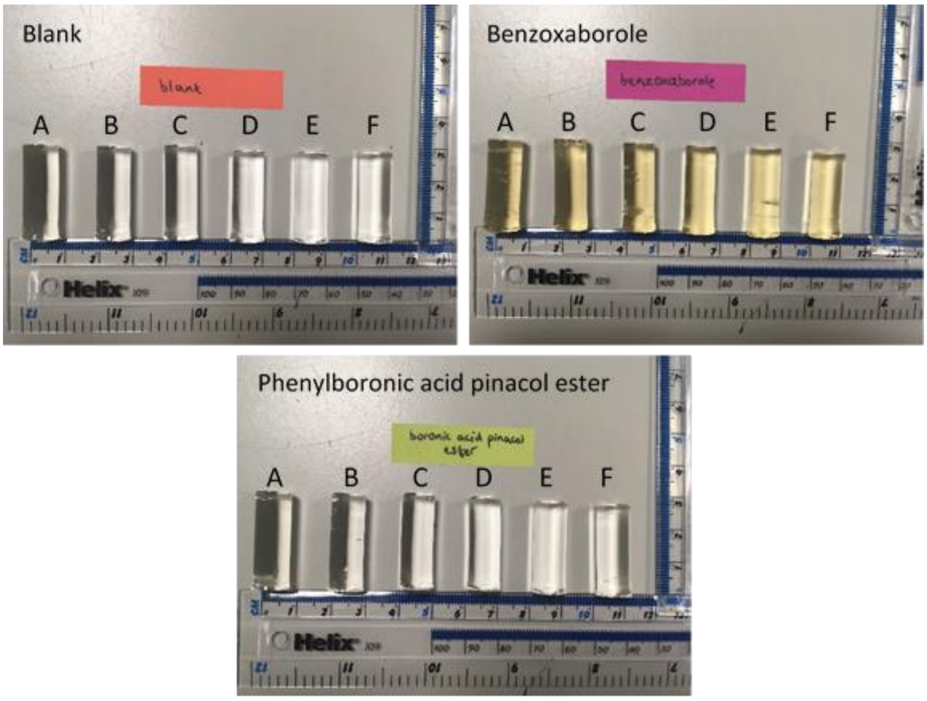

Figure S4 -Photograph of hydrogels before standing in (a) pure water, (b) pH 7.3 phosphate buffer, (c) pH 8.5 phosphate buffer, (d) pH 10 phosphate buffer, (e) pH 8.21 phosphate buffer (52.1 wt % MeOH), (f) pH 10 phosphate buffer (52.1 wt % MeOH).

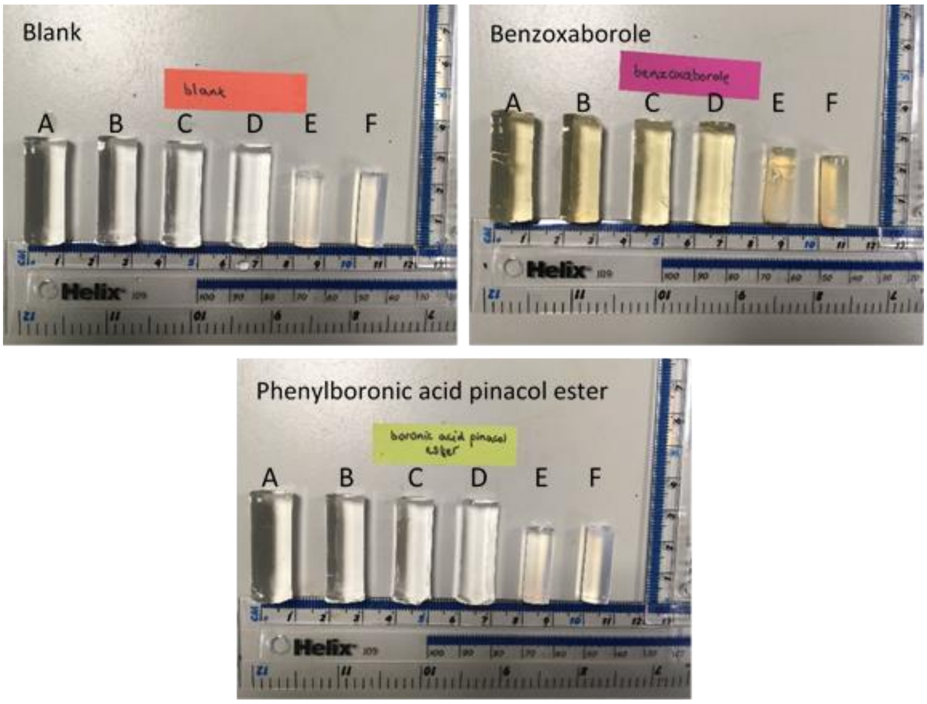

Figure S5 - Photograph of hydrogels after overnight standing in (a) pure water, (b) pH 7.3 phosphate buffer, (c) pH 8.5 phosphate buffer, (d) pH 10 phosphate buffer, (e) pH 8.21 phosphate buffer (52.1 wt % MeOH), (f) pH 10 phosphate buffer (52.1 wt % MeOH)

3. Quantitative data

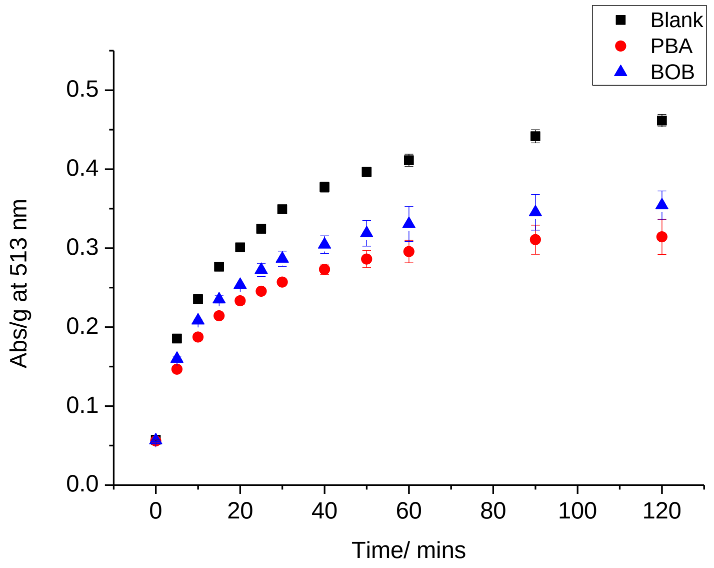

Figure S6 - UV absorption per unit mass of hydrogels (at 513 nm) for dye release in PBS (pH 7.3)

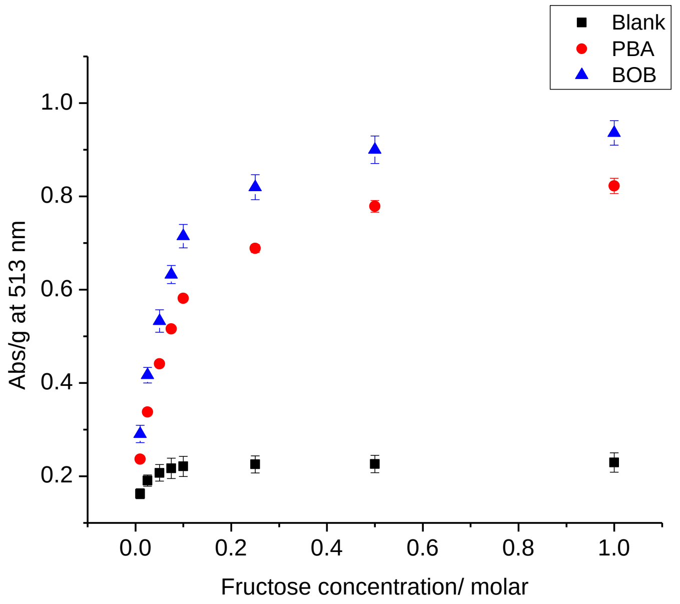

Figure S7 - UV absorption per unit mass of hydrogels (at 513 nm) with addition of fructose in PBS (pH 7.3)

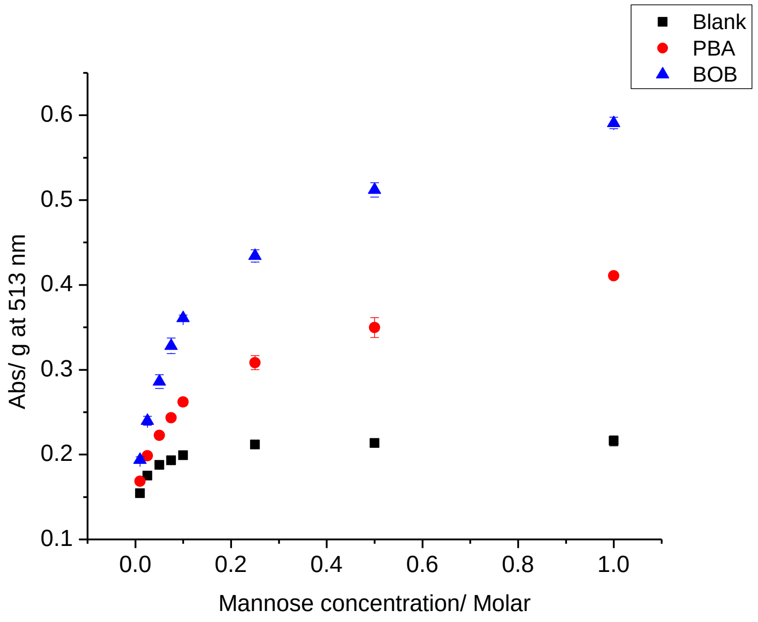

Figure S8 - UV absorption per unit mass of hydrogels (at 513 nm) with addition of mannose in PBS (pH 7.3)

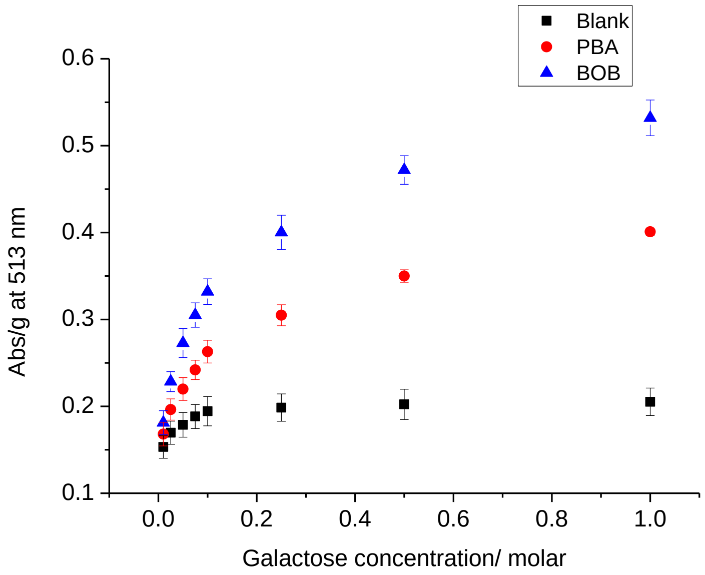

Figure S9 - UV absorption per unit mass of hydrogels (at 513 nm) with addition of galactose in PBS (pH 7.3)

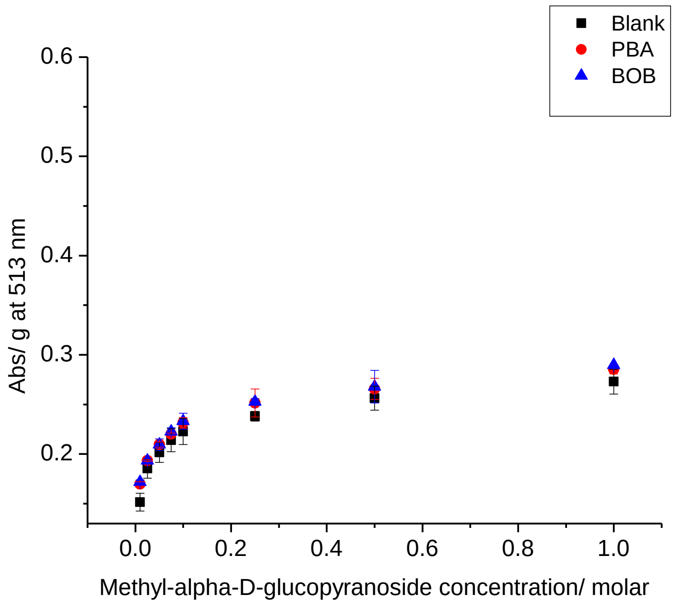

Figure S10 - UV absorption per unit mass of hydrogels (at 513 nm) with addition of methyl α-D-glucopyranoside in PBS (pH 7.3)

Table S4. Relative amount of dye release (abs/g) with addition of various saccharides (1 M)

Gel	Fructose	Galactose	Mannose	Glucose
BOB	0.66[a]	0.35[a]	0.41[a]	0.33[a]
PBA	0.57[a]	0.23[a]	0.25a]	0.18[a]
Blank	0.07[a]	0.06[a]	0.05a]	0.05[a]

[a] Abs / g at 513 nm for 1 M saccharide minus the Abs / g at 513 nm for 0 mM saccharide

4. Limit of Detection

The limit of detection was calculated using the formula shown below:

$$\text{Limit of detection} = 3\sigma/K$$

Where σ is the standard deviation of blank measurement, and K is the slope of the absorbance *versus* saccharide concentration.

LOD: Glucose

BOB - 0.018 M

PBA - 0.031 M

LOD: Mannose

BOB 0.012 M

PBA 0.017 M

LOD: Fructose

BOB 0.016 M

PBA 0.021 M

LOD: Galactose

BOB 0.020 M

PBA 0.016 M

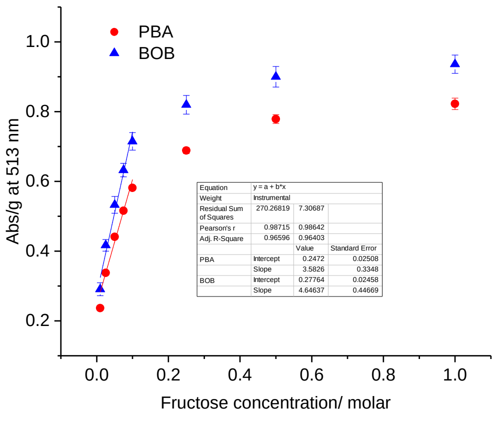

Equation	y = a + b*x		
Weight	Instrumental		
Residual Sum of Squares	270.26819	7.30687	
Pearson's r	0.98715	0.98642	
Adj. R-Square	0.96596	0.96403	
		Value	Standard Error
PBA	Intercept	0.2472	0.02508
	Slope	3.5826	0.3348
BOB	Intercept	0.27764	0.02458
	Slope	4.64637	0.44669

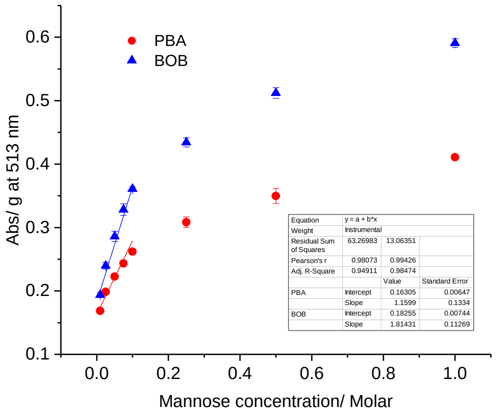

Equation	y = a + b*x		
Weight	Instrumental		
Residual Sum of Squares	63.26983	13.06351	
Pearson's r	0.98073	0.99426	
Adj. R-Square	0.94911	0.98474	
		Value	Standard Error
PBA	Intercept	0.16305	0.00647
	Slope	1.1599	0.1334
BOB	Intercept	0.18255	0.00744
	Slope	1.81431	0.11269

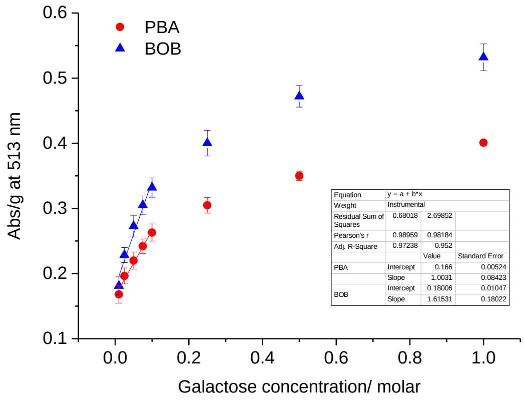

Equation	y = a + b*x		
Weight	Instrumental		
Residual Sum of Squares	0.68018	2.69852	
Pearson's r	0.98959	0.98184	
Adj. R-Square	0.97238	0.952	
		Value	Standard Error
PBA	Intercept	0.166	0.00524
	Slope	1.0031	0.08423
BOB	Intercept	0.18006	0.01047
	Slope	1.61531	0.18022

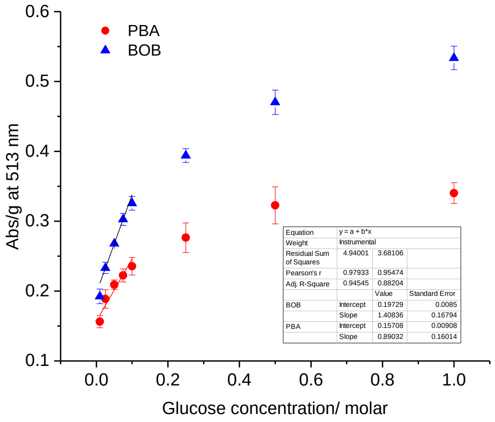

Equation	y = a + b*x		
Weight	Instrumental		
Residual Sum of Squares	4.94001	3.68106	
Pearson's r	0.97933	0.95474	
Adj. R-Square	0.94545	0.88204	
		Value	Standard Error
BOB	Intercept	0.19729	0.0085
	Slope	1.40836	0.16794
PBA	Intercept	0.15708	0.00908
	Slope	0.89032	0.16014

5. Experimental

Benzo[c][1,2]oxaborol-1(3H)-ol

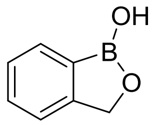

2-formylphenylboronic acid (10.00 g, 66.69 mmol) was dissolved in methanol (100 mL). $NaBH_4$ (2.78 g, 73.36 mmol) was added in portions and the resulting solution was left to stir at room temperature for 1 hour. The reaction mixture was acidified with 3 M HCl and the aqueous phase was extracted into diethyl ether 3 times. The combined organic layers were dried over $MgSO_4$, filtered, and the solvent was removed under reduced pressure to give a white powdery solid in 99.8% yield. ^{1}H NMR (500 MHz, DMSO-d_6): δ 9.17 (1H, OH, s (br)) 7.74 (1H, ArH, d, J = 7.3 Hz), 7.47 (1H, ArH, t, J = 7.5 Hz), 7.40 (1H, ArH, d, J = 7.5 Hz), 7.34 (1H, ArH, t, J = 7.1 Hz), 4.98 (2H, CH_2, s); ^{13}C NMR (75 MHz, DMSO-d_6): δ 154.22, 130.86, 130.83, 127.15, 121.70, 70.27 ^{11}B NMR (96 MHz, DMSO-d_6): δ 35.54; FTIR ν (cm^{-1}): 3291, 3212, 3052, 968; HRMS (ES) *m/z* calculated for $C_7H_6O_2B_1$: $[M-H]^-$ 133.0461, found 133.0476; Mp: 96-98 ℃ (lit. 96-99 ℃)[2]

6-Nitrobenzo[c][1,2]oxaborol-1(3H)-ol

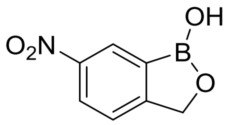

To 40 mL fuming nitric acid maintained at -30 to -40 °C, using dry ice and acetone, benzoxaborole (8.00 g, 59.73 mmol) was added in portions with stirring. N_2 gas was flowed over the top of the reaction flask during addition to stop atmospheric air condensing inside. The reaction mixture was maintained at -30 to -40 °C and left to stir for 1 hour. The reaction mixture was poured onto ice water and left to stir until the ice had melted. The precipitated pale yellow compound was filtered, washed with water and dried under vacuum to give a pale yellow powdery solid. The crude reaction product was recrystallized from hot EtOH. The purified product was collected as a pale yellow powder in 66% yield. ^{1}H NMR (300 MHz, DMSO-d_6): δ 9.56 (1H, OH, s (br)), 8.58 (1H, ArH, d, J = 2.2 Hz), 8.33 (1H, ArH, dd, J_1 = 8.4, J_2 = 2.4 Hz), 7.69 (1H, ArH, d, J = 8.9 Hz), 5.13 (2H, CH_2, s); ^{13}C NMR (75 MHz, DMSO-d_6): δ 160.96, 147.51, 125.97, 125.84, 123.41, 70.43; ^{11}B NMR (96 MHz, DMSO-d_6): δ 33.69; FTIR ν (cm^{-1}): 3257, 3072, 1512, 1339, 979; HRMS (ES) *m/z* calculated for $C_7H_6N_1O_4B_1Na_1$: $[M+Na]^+$ 202.0289, found 202.0279; Mp: 176-179 °C

6-Aminobenzo[c][1,2]oxaborol-1(3H)-ol

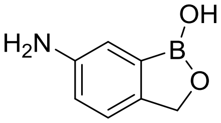

6-Nitrobenzoxaborole (2.00 g, 11.18 mmol) was dissolved in 10% acetic acid/THF (40 mL). Pd/C (1.19 g, 11.18 mmol) was added in portions, followed by stirring at room temperature for 24 hours with continuously bubbled H_2 under atmospheric pressure. The reaction mixture was purged with N_2 for a few minutes. After removing Pd/C by filtration through celite, the filtrate was evaporated to a brown oil under reduced pressure. The product was resolved in EtOAc (50 mL) and treated with 1 M aq. LiOH (100 mL) at room temperature for 30 minutes. The EtOAc layer was separated out and washed 3 times with 1 M LiOH. The alkaline layers were combined, acidified to pH 6-7 with 3 M aq. HCl and extracted 5 times into EtOAc. The organic extracts were combined, dried over $MgSO_4$, filtered, and the solvent was removed under reduced pressure to give a brown solid in 78% yield. ^{1}H NMR (300 MHz, DMSO-d_6): δ 8.89 (1H, OH, s), 7.03 (1H, ArH, d, J = 8.6 Hz), 6.88 (1H, ArH, d, J = 1.9 Hz), 6.70 (1H, ArH, dd, J_1 = 8.2, J_2 = 2.2 Hz), 4.96 (2H, NH_2, s (br)), 4.81 (2H, CH_2, s); ^{13}C NMR (75 MHz, DMSO-d_6): δ 147.93, 141.70, 121.76, 117.90, 114.90, 69.96; ^{11}B NMR (96 MHz, DMSO-d_6): δ 36.01; FTIR ν (cm^{-1}): 3352, 2926, 2874, 1621, 981; HRMS (ES) m/z calculated for $C_7H_9N_1O_2B_1$: $[M+H]^+$ 150.0726, found 150.0739; Mp: 142-144 ℃

***N*-(1-Hydroxy-1,3-dihydrobenzo[c][1,2]oxaborol-6-yl)methacrylamide**

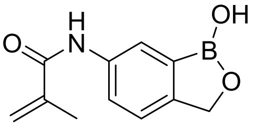

6-Aminobenzoxaborole (1.00 g, 6.71 mmol) and sodium hydrogen carbonate (2.26 g, 26.84 mmol) were dissolved in a 1:1 mixture of THF (20 mL) and water (20 mL). The resulting solution was cooled down to 0 ℃ and methacryloyl chloride (1.32 mL, 13.42 mmol) was added dropwise. The reaction was kept at 0 ℃ for 2 hours and was then allowed to warm to room temperature and stirred overnight. A solid crude product was obtained after THF was evaporated under reduced pressure, and extracted into EtOAc for 2 hours. The EtOAc layer was washed continuously with water, saturated sodium bicarbonate solution, water, and brine. The organic layer was dried over $MgSO_4$, filtered and the solvent was removed under reduced pressure to give a pale brown solid in 82% yield. ^{1}H NMR (300 MHz, DMSO-d_6): δ 9.81 (1H, NH, s), 9.19 (1H, OH, s), 8.07 (1H, ArH, d, J = 1.6 Hz), 7.68 (1H, ArH, dd, , J_1 = 8.2, J_2 = 1.9 Hz), 7.35 (1H, ArH, d, J = 8.2 Hz), 5.82 (1H, CH, s), 5.51 (1H, CH, s), 4.95 (2H, CH_2, s), 1.97 (3H, CH_3, s); ^{13}C NMR (75 MHz, DMSO-d_6): δ 167.10, 149.34, 140.77, 138.12, 123.85, 122.60, 121.68, 120.20, 70.05, 18.46; ^{11}B NMR (96 MHz, DMSO-d_6): δ 23.76; FTIR ν (cm^{-1}): 3276, 3086, 2973, 1655, 1214, 979; HRMS (ES) *m/z* calculated for $C_{11}H_{13}N_1O_3B_1$: $[M+H]^+$ 218.0988, found 218.0993; Mp: 167-170 ℃

6. NMR

Benzo[c][1,2]oxaborol-1(3H)-ol ^{1}H NMR (500 MHz, DMSO-d_6)

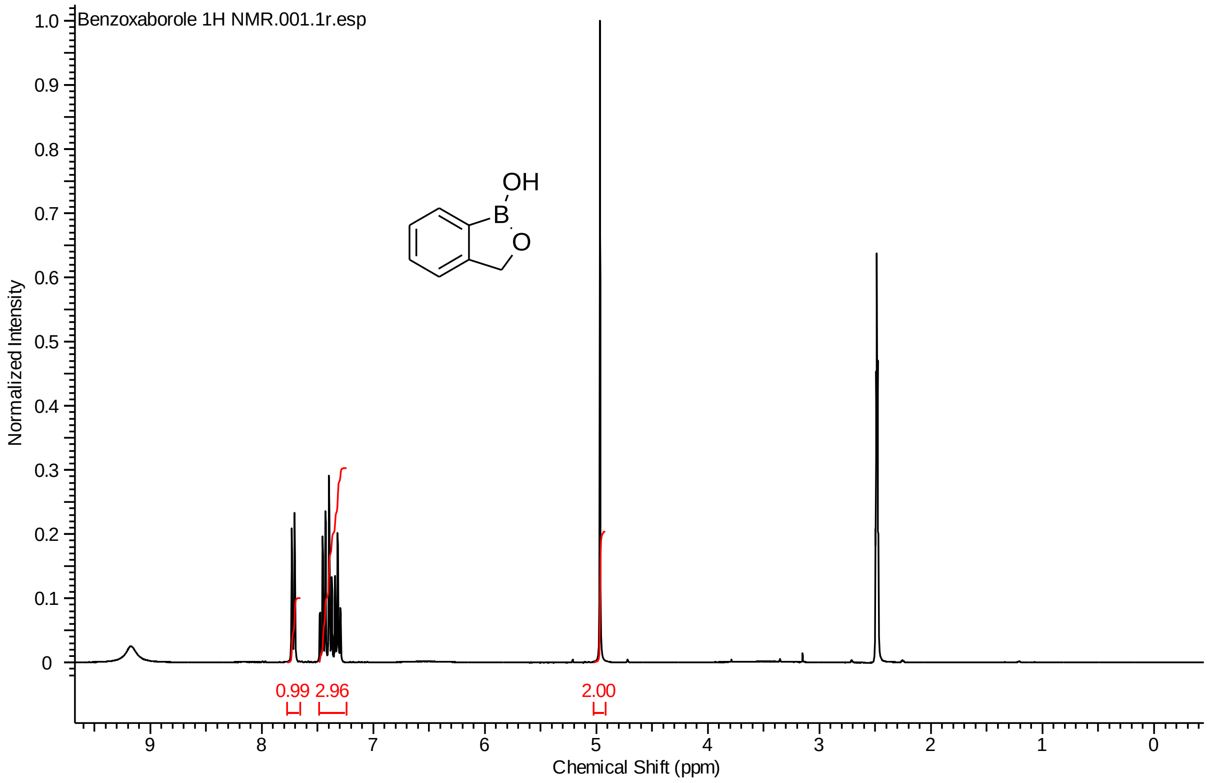

Benzo[c][1,2]oxaborol-1(3H)-ol ^{13}C NMR (75 MHz, DMSO-d_6)

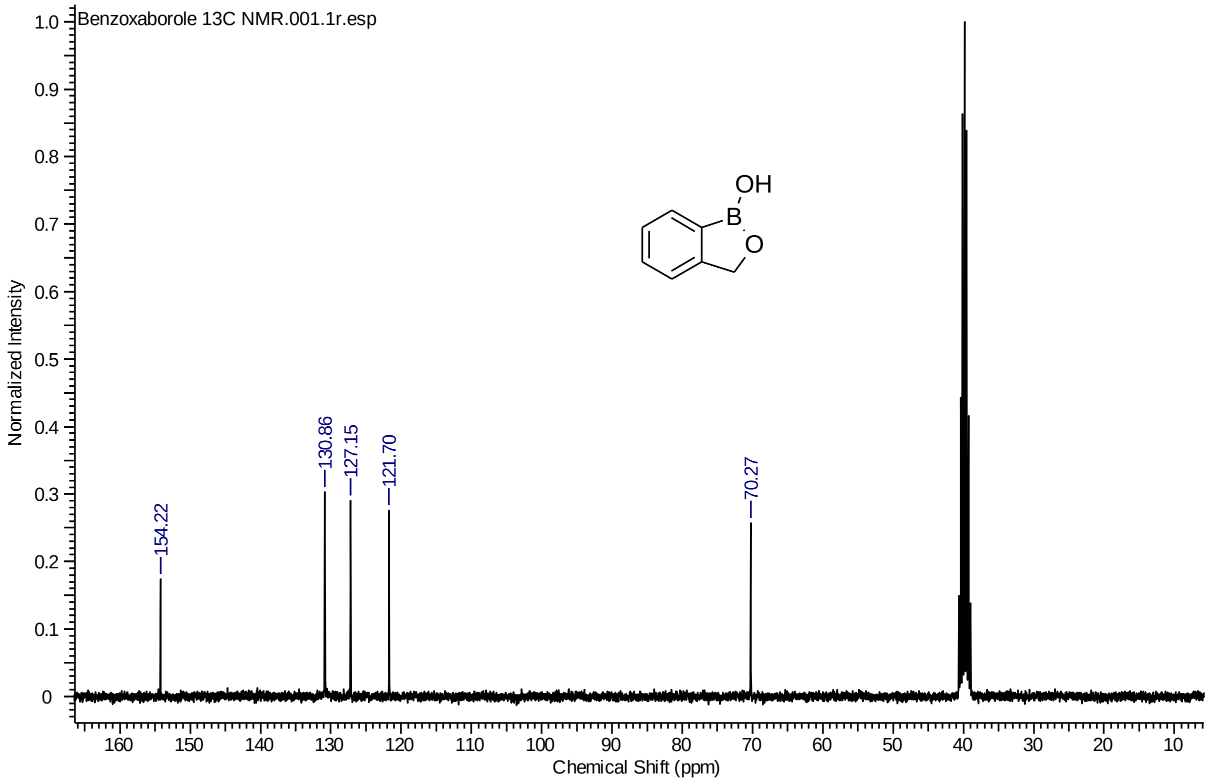

6-Nitrobenzo[c][1,2]oxaborol-1(3H)-ol ^{1}H NMR (300 MHz, DMSO-d_6)

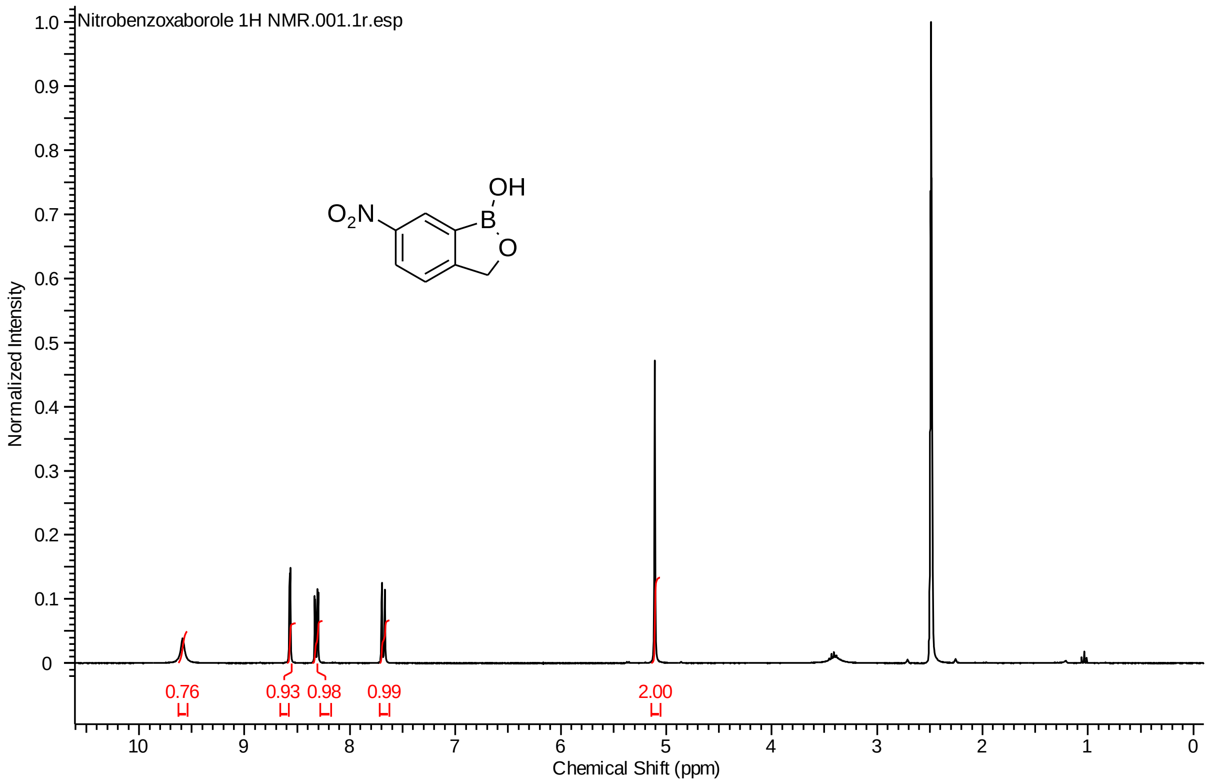

6-Nitrobenzo[c][1,2]oxaborol-1(3H)-ol ^{13}C NMR (75 MHz, DMSO-d_6):

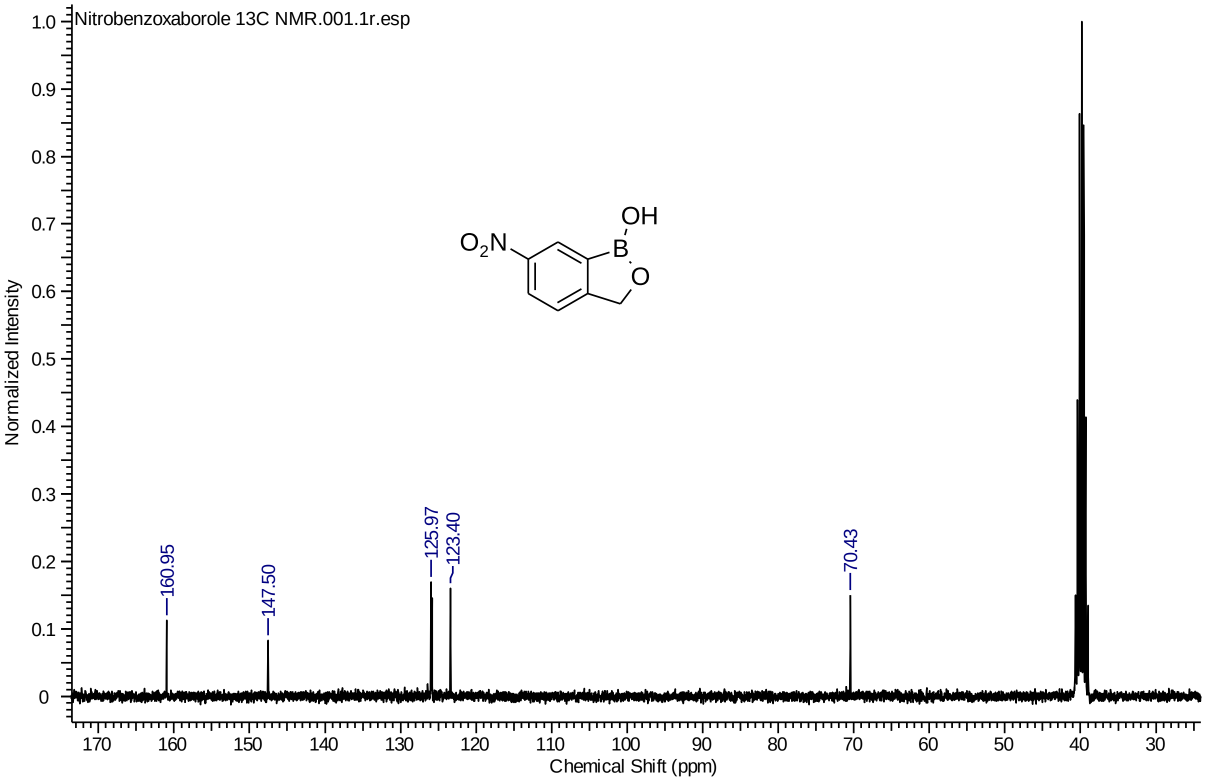

6-Aminobenzo[c][1,2]oxaborol-1(3H)-ol (300 MHz, DMSO-d_6)

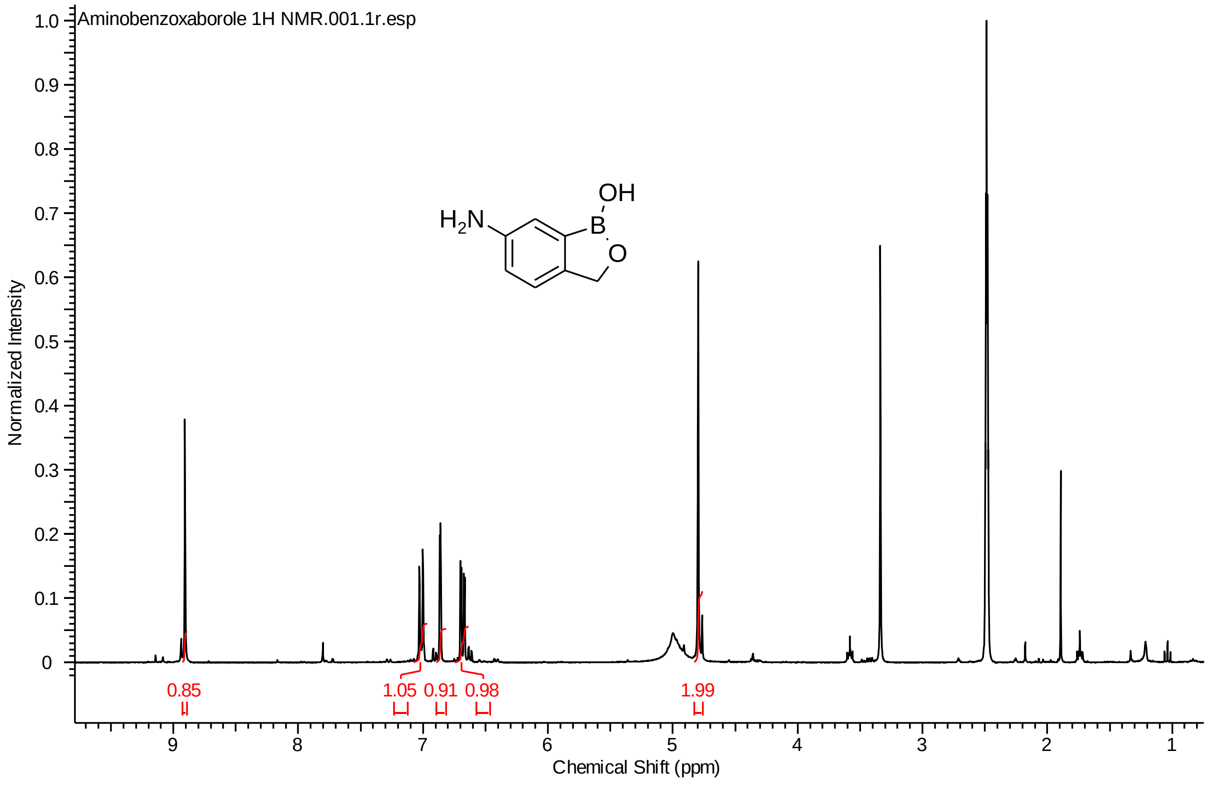

6-Aminobenzo[c][1,2]oxaborol-1(3H)-ol ^{13}C NMR (75 MHz, DMSO-d_6)

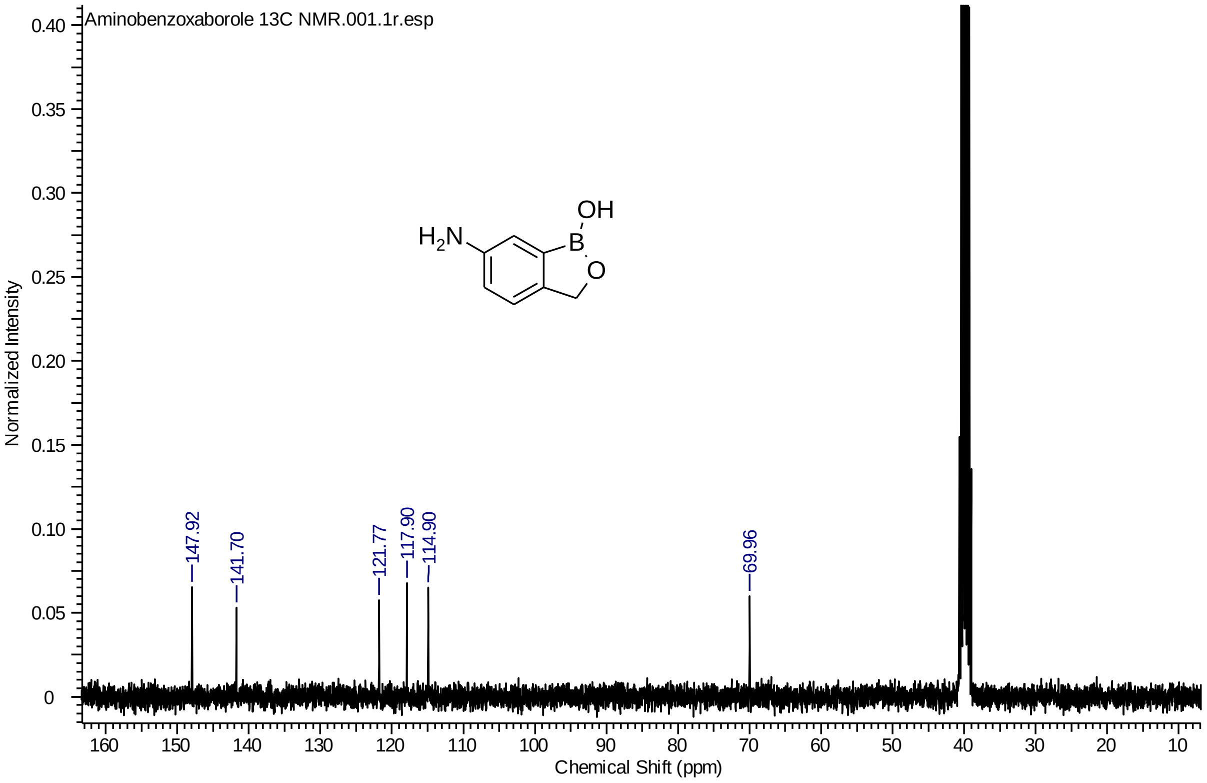

***N*-(1-Hydroxy-1,3-dihydrobenzo[c][1,2]oxaborol-6-yl)methacrylamide** (300 MHz, DMSO-d_6)

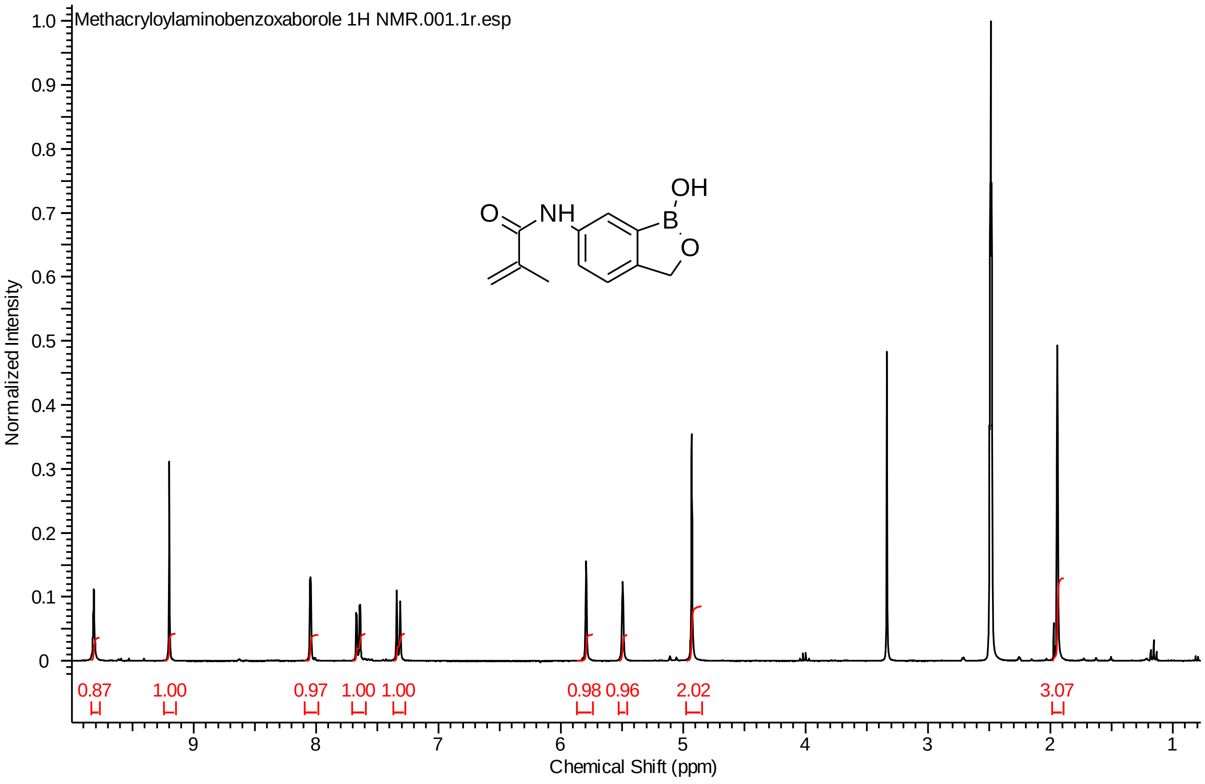

***N*-(1-Hydroxy-1,3-dihydrobenzo[c][1,2]oxaborol-6-yl)methacrylamide** ^{13}C NMR (75 MHz, DMSO-d_6)

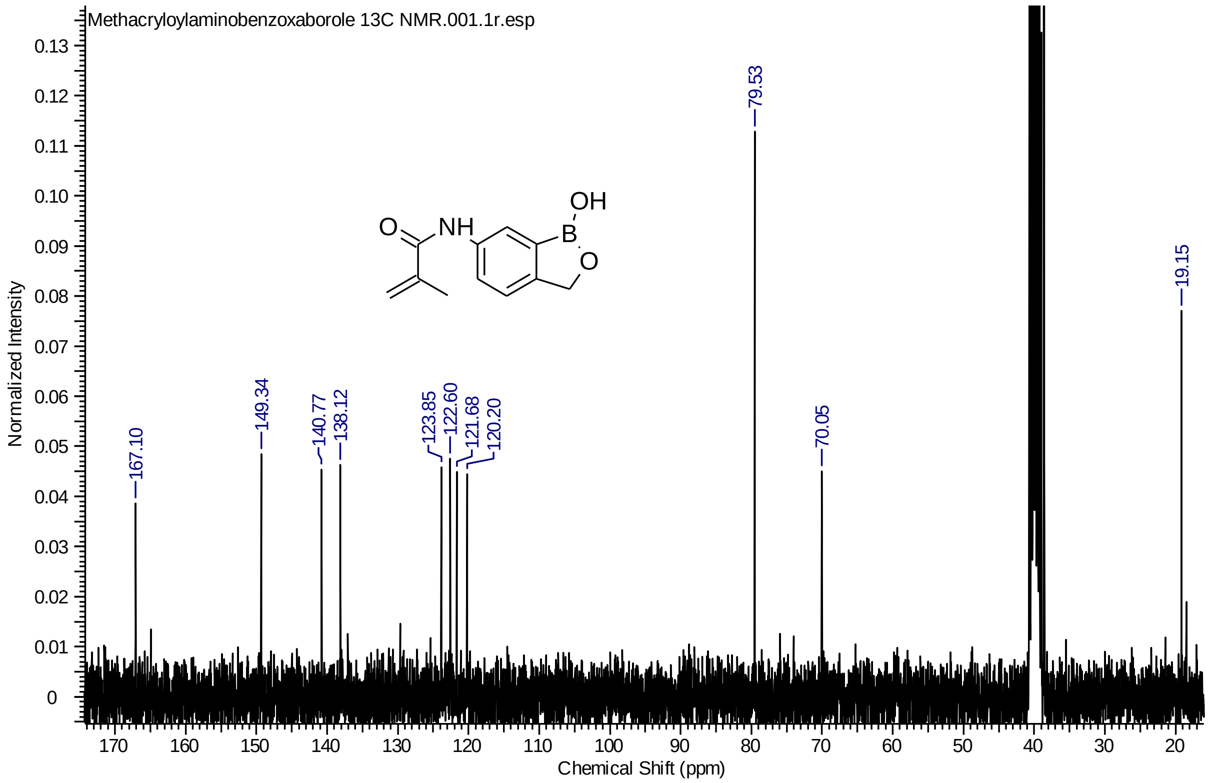

Supplement: Supplementary file 1 — Supplementary [file OPEN-7-266-s001.pdf]
